# Cystic adventitial disease of the popliteal artery: report of two cases

**DOI:** 10.1007/s00595-013-0675-5

**Published:** 2013-08-01

**Authors:** Hua Zhang, Yang Zhang, Qi Wang, Wen-Guang Zhao, Jia-Ju Wang

**Affiliations:** Department of Vascular Surgery, The First Affiliated Hospital of Jilin University, 908 Mingde Road, Chaoyang District, Changchun, 130021 Jilin Province China

**Keywords:** Cystic adventitial disease, Popliteal artery, Surgical resection, Bypass grafting

## Abstract

Cystic adventitial disease (CAD) of the popliteal artery is a rare vascular disorder in which a mucin-containing cyst develops in the adventitial layer of the artery. We report two such cases, each of which was treated differently. The first case was of a 49-year-old man, treated by excision of the involved arterial segment and interposition of an autologous saphenous vein graft. The second case was of a 36-year-old man, treated by local excision of the affected arterial segment and interposition with prosthetic bypass grafting. Both patients presented with rapidly progressing intermittent claudication of the lower extremities, but without remarkable evidence of atherosclerotic disease. Physical examination revealed diminished or absent popliteal, posterior tibial and dorsalis pedis pulses in the lower extremities. Color Doppler ultrasound of the popliteal artery revealed hypoechoic cystic lesions surrounding the vessel, and popliteal arterial stenosis, in both patients. Surgery resulted in immediate improvement of the arterial pulse distal to the lesion. Both patients recovered uneventfully. Thus, resection of the involved artery segment and interposition bypass grafting, using either patient or prosthetic veins, offers favorable results for CAD of the popliteal artery.

## Introduction

Cystic adventitial disease (CAD) is an uncommon non-atherosclerotic condition in which a cystic collection of mucinous material accumulates in the adventitial layer of the artery. The subsequent extrinsic compression of the arterial lumen leads to peripheral vascular insufficiency and intermittent claudication. CAD most often affects the popliteal artery, with 85 % of documented cases involving this region. Other involved arterial segments that have been reported include the external iliac, common femoral, radial, and ulnar arteries. CAD patients are typically young to middle-aged men, with a male-to-female ratio of approximately 15:1 and an average age in the fourth to fifth decade of life [1]. Since the disease often progresses rapidly, early diagnosis and prompt initiation of treatment, prior to arterial occlusion occurring, is important to lower morbidity. This report describes two cases of successful enucleation of involved arterial segments and interposition via the patient vein or prosthetic bypass grafting. We also review the general pathogenesis, clinical features, and management of CAD of the popliteal artery.

## Case report

### Patient 1

**Fig. 1:**
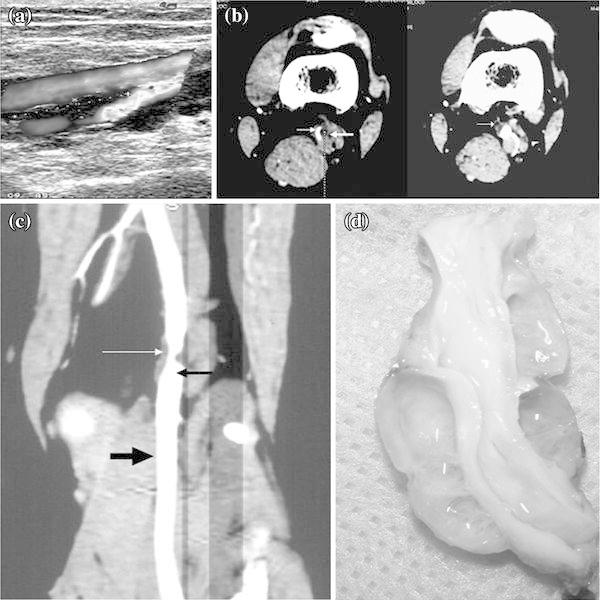
Case 1: a 49-year-old man with cystic adventitial disease (CAD) of the popliteal artery underwent surgical excision of the cyst and interposition saphenous vein bypass grafting. **a** Color Doppler ultrasound showed a hypoechoic cystic mass that was anterolateral to the vessel and resulted in endoluminal stenosis. **b** Axial computed tomography (CT) images showed a hypoattenuating mass (*thin arrowhead*) causing extrinsic compression of the popliteal artery with resultant arterial stenosis (*thick arrowhead*). **c** CT angiography showed a hypoattenuating mass resulting in endoluminal compression (*white arrowhead*) with a normal lumen in the distal and proximal artery (*black arrowhead*). **d** The excised cyst was a multilobulated pinkish-white lesion with a firm wall

A 49-year-old man presented with a 3-month history of intermittent claudication of the left lower extremity. Physical examination revealed diminished popliteal, posterior tibial, and dorsalis pedis pulses in the left lower extremity and a systolic murmur in the popliteal fossa. Color Doppler ultrasound showed a hypoechoic cystic mass anterolateral to the popliteal artery and popliteal arterial stenosis (Fig. 1a). An axial computerized tomography (CT) scan showed extrinsic compression of the popliteal artery, caused by the cysts (Fig. 1b). CT angiography showed that external compression of the popliteal artery by hypoattenuating cystic masses resulted in hemodynamically significant endoluminal stenosis with normal lumina of the proximal and distal arteries (Fig. 1c).

At surgery, the popliteal artery was exposed from a posterior approach through a longitudinal incision, revealing a firm, pinkish-white, multilobulated cystic lesion, with well-defined margins (Fig. 1d). There appeared to be no communication between the cystic lesions and the joint. We excised the adventitial cyst with the affected artery completely. No intravascular thrombus was found, but the involved arterial wall had obvious uneven thickness, resulting in some extent of arterial stenosis. In situ vascular reconstruction was performed using a homolateral great saphenous vein graft. Immediately after the operation, normal popliteal and distal pedal pulses were palpated. The ankle brachial indexes were 0.72 and 1.16 preoperatively and postoperatively, respectively.

### Patient 2

**Fig. 2:**
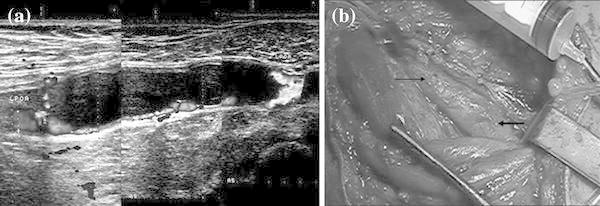
Case 2: a 36-year-old man with CAD of the popliteal artery underwent surgical excision of the cyst and interposition prosthetic bypass grafting. **a** Color Doppler ultrasound showed a cystic mass posterior to the popliteal artery causing endoluminal stenosis. Only a slim blood signal was observed between the cystic mass and the popliteal anterior wall. **b** Intraoperatively, the involved popliteal artery exhibited a cystic protuberance (*thin arrowhead*) with a normal proximal arterial lumen (*thick arrowhead*)

A 36-year-old man presented with a 2-year history of a cold left foot and left calf pain after exercise, and reported that these symptoms had worsened over the past month. Physical examination revealed mild amyotrophia and a 7 °C difference between the thigh and calf skin on the left leg. No pulse was palpated below the popliteal artery on the left side. Color Doppler ultrasound showed a multilobulated cystic mass, 40 mm × 11 mm in size, which was posterior to the popliteal artery and resulted in hemodynamically significant endoluminal stenosis. Only a thin linear arterial blood flow signal of 0.9–1.1 mm thickness was observed inside the affected popliteal artery, caused by compression from the cystic mass (Fig. 2a).

At surgery, a cystic protuberance, 6 cm long, was found at the junction of the femoral and popliteal artery (Fig. 2b). The translucent adventitia of the popliteal artery was opened longitudinally, and the intact arterial intima was found to have significant local thickening. A cystic collection of gelatinous material had accumulated within the adventitia of the popliteal artery, which resulted in significant endoluminal stenosis. The cystic lesion was removed completely using a conservative approach to maintain the medial and intimal layers; however, this did not improve the arterial stenosis remarkably. The patient had a severe form of bilateral varicose veins and assuming that the diseased vein was implanted into the arterial system, the thin vein wall would have been subjected to the high arterial shear pressure. As the resulting elevated vein pressure could lead to the formation of an aneurysm of the thin wall, we did not use the saphenous vein as the interposition graft. In situ vascular reconstruction was performed by a prosthetic ePTFE graft. Immediately after the operation, normal popliteal and distal pedal pulses were felt. The ankle brachial index increased from 0.46 to 0.97 after the successful vascular reconstruction.

## Discussion

CAD is a rare disease characterized by a mucoid cyst in the adventitia, which compresses the affected artery and restricts blood flow. This entity was first reported by Atkins and Key [2], who described CAD affecting the external iliac artery. CAD of a popliteal artery was first described by Ejrup and Hiertonn [3], who reported the case of a patient who had acute ischemia onset and intermittent claudication. CAD cases reported since then have indicated a predilection for the popliteal artery. The current incidence of CAD is estimated to be 1 in every 1200 cases of calf claudication [1].

The etiology of CAD is still not completely understood, although four theories have been proposed [4, 5]. The first theory attributes CAD to a myxomatous systemic degenerative condition associated with a generalized disease. Second, it is presumed that knee flexion frequently stretches or distorts the proximity of the popliteal artery; thus, repeated trauma to the adventitia might cause destruction and promote cystic degeneration of the adventitial wall. Third, adventitial cysts are known to arise from synovial ganglia that migrate into the adventitia of the affected vessel via direct communication with the synovial structures of the adjacent joint. Fourth, during the embryologic development of the vessel, undifferentiated mesenchymal cells are incorporated into the arterial wall. It is these mucin-secreting mesenchymal cells that will subsequently produce mucoid materials, from which the adventitial cysts will originate. The last two hypotheses are considered by many as the most reasonable for explaining CAD.

CAD of the popliteal artery generally occurs in males during the fourth and fifth decades of life. The CAD cysts progressively compress the lumen of the popliteal artery, which manifests as intermittent claudication. The patient will experience this symptom with sudden onset and rapid progression. Thus, when a middle-aged adult presents with intermittent claudication in the lower extremities and no evidence of atherosclerotic disease, the treating physician should consider CAD of the popliteal artery. On physical examination, the popliteal and pedal pulses can be normal or weak; therefore, gray-scale ultrasound is often used to identify the suspected cysts as hypoechoic or anechoic masses adjacent to the affected vessel, especially since this imaging modality is noninvasive and readily available in many clinics [6]. Color Doppler ultrasound is slightly more powerful as it can reveal arterial stenosis or occlusion, as evidenced by a dim or absent blood signal [6]. CT and magnetic resonance imaging (MRI) are even more informative because they clearly depict the anatomy of the popliteal fossa and can show the anatomical relationship between the cysts and vessels simultaneously [7, 8]. CT images identify the cysts as hypoattenuating masses in the arterial wall and an intravenous contrast agent will cause a rim of enhancement on the CT image in the thin cystic wall with no enhancement of the mucinous content of the cyst. On MRI, the cysts are hyperintense on T_2_-weighted images and have low to intermediate signal intensity on T_1_-weighted images, caused by the variable amount of mucoid material within the cysts [9]. Cyst-mediated compression of the popliteal artery can be seen on axial MR or CT images. CAD can also be identified as distinctive stenosis of the popliteal arterial lumen by arteriography. If the cysts are concentric, the luminal stenosis will have an hour-glass appearance, whereas if they are eccentric, the stenosis will exhibit the typical scimitar sign. However, arteriography is not recommended as a routine procedure because of its invasiveness and reliance on ionizing radiation exposure.

Three treatment options are currently recommended for CAD: resection of the cyst with arterial preservation, excision of the involved arterial segment with interposition bypass grafting, and CT- or ultrasound-guided percutaneous cyst aspiration. The treatment of choice is surgical removal of the cyst, which achieves complete excision of the cyst wall and preserves the medial and intimal layers. This approach is most suitable for patients without severe popliteal stenosis and minimal adherence between the cyst wall and the artery [9]. In cases where the joint and cysts are communicating, it is generally recommended that the two should be removed together [10]. Cases of severe arterial stenosis or occlusion necessitate interposition bypass grafting, especially when extrinsic compression has been released [6]. Although ultrasound- or CT-guided aspiration has been used successfully to treat CAD of the popliteal artery [8, 11], the reported frequency of recurrence after aspiration is high. It is believed that the high viscosity of the mucinous and gelatinous cyst content inhibit complete removal by the aspiration process. There is one report of endovascular stenting being used to treat popliteal artery stenosis that arose secondary to CAD with significant claudication. However, despite technical success, the implanted stent remained patent for only 1 week and the patient eventually underwent interposition venous graft reconstruction to resolve the CAD [12].

In conclusion, CAD should be suspected in middle-aged male patients who present with intermittent claudication of lower extremities but without solid evidence of atherosclerotic disease. Ultrasound, CT and MR imaging modalities are valuable tools for acquiring a correct diagnosis of CAD. When surgical removal of the CAD does not release the popliteal arterial stenosis, excision of the involved arterial segment and interposition bypass grafting produces excellent results.
